# Gene dispersion is the key determinant of the read count bias in differential expression analysis of RNA-seq data

**DOI:** 10.1186/s12864-017-3809-0

**Published:** 2017-05-25

**Authors:** Sora Yoon, Dougu Nam

**Affiliations:** 1 School of Life Sciences, Ulsan National Institute of Science and Technology, Ulsan, Republic of Korea; 2 Department of Mathematical Sciences, Ulsan National Institute of Science and Technology, Ulsan, Republic of Korea

**Keywords:** RNA-seq, Differential expression analysis, Read count bias, Gene length bias, Dispersion

## Abstract

**Background:**

In differential expression analysis of RNA-sequencing (RNA-seq) read count data for two sample groups, it is known that highly expressed genes (or longer genes) are more likely to be differentially expressed which is called *read count bias* (or gene length bias). This bias had great effect on the downstream Gene Ontology over-representation analysis. However, such a bias has not been systematically analyzed for different replicate types of RNA-seq data.

**Results:**

We show that the dispersion coefficient of a gene in the negative binomial modeling of read counts is the critical determinant of the read count bias (and gene length bias) by mathematical inference and tests for a number of simulated and real RNA-seq datasets. We demonstrate that the read count bias is mostly confined to data with small gene dispersions (e.g., technical replicates and some of genetically identical replicates such as cell lines or inbred animals), and many biological replicate data from unrelated samples do not suffer from such a bias except for genes with some small counts. It is also shown that the sample-permuting GSEA method yields a considerable number of false positives caused by the read count bias, while the preranked method does not.

**Conclusion:**

We showed the small gene variance (similarly, dispersion) is the main cause of read count bias (and gene length bias) for the first time and analyzed the read count bias for different replicate types of RNA-seq data and its effect on gene-set enrichment analysis.

**Electronic supplementary material:**

The online version of this article (doi:10.1186/s12864-017-3809-0) contains supplementary material, which is available to authorized users.

## Background

High-throughput cDNA sequencing (RNA-seq) provides portraits of the transcriptome landscape at an unprecedented resolution [1, 2]. RNA-seq typically produces millions of sequencing reads, each of which provides a bit of information for genomic events in the cell. Thus, unlike microarray, RNA-seq has diverse applications for genomic analyses such as quantification of gene expression, finding of new transcripts, detection of single nucleotide polymorphisms, RNA editing, gene fusion detection and so on [3–8]. Among these applications, the quantification of gene expression may be a key function of RNA-seq. It is performed by simply counting the reads aligned to each gene or exon region. RNA-seq also has advantages in this application over microarray in both the reproducibility and the sensitivity in detecting weakly expressed transcripts [9].

Molecular biological research has focused on questions such as ‘what happens in the cell’ and ‘what changes between differing cell conditions’. While the sequencing technology has shown advantages for answering the former question, the latter gave rise to some complicated issues as follows: (1) *normalization*: In contrasting RNA-seq counts between different cell conditions, each sample can have different sequencing depths and RNA compositions. Therefore, appropriate normalization should be applied to make the gene expression levels comparable or to estimate the model parameters [10–12]. (2) *probability modelling*: Since they are counting data, discrete probability models (Poisson or negative binomial model) have been used to test the differential expression (DE) of genes. Parameter estimation is a critical issue especially for data with small replicates [9, 13, 14]. (3) *biases in DE analysis*: striking biases with DE analysis of RNA-seq count data were found in that highly expressed genes or long genes had a greater likelihood of being detected to be differentially expressed, which are called the *read count bias* and *gene length bias*, respectively [15]. These biases hampered the downstream Gene Ontology over-representation analysis (denoted by *GO analysis*) such that GO terms annotated to many long genes had a greater chance of being selected. A resampling based method was eventually developed to account for the selection bias in GO analysis [16] and followed by other approaches [17, 18]. Because the read count bias and gene length bias represent virtually the same type of bias, we will mainly focus on the read count bias and add some result for the gene length bias. Despite the profound effect that the read count bias might have on DE and the downstream functional analyses, it has been witnessed that some RNA-seq datasets do not suffer from such a bias which necessitates further investigation [19, 20]. Note that the gene length bias was originally shown for the simple *Poisson* model and mostly for the technical replicate data [15]. Thus, such a bias needs to be further analyzed for over-dispersed *Poisson* model (negative binomial) and biological replicate data.

In this study, it is shown that the gene dispersion value as estimated in the negative binomial modelling of read counts [13, 14] is the key determinant of the read count bias. We found that the read count bias in DE analysis of RNA-seq data was mostly confined to data with small gene dispersions such as technical replicate or some of the *genetically identical* (GI) replicate data (generated from cell lines or inbred model organisms). In contrast, the replicate data from unrelated individuals, denoted by *unrelated replicates*, had overall tens to hundreds times greater gene dispersion values than those of technical replicate data, and DE analysis with such unrelated replicate data did not exhibit the read count bias except for genes with some small read counts (< tens). Such a pattern was observed for different levels of DE fold changes and sequencing depths. Although DE analysis of technical replicates is not meaningful, it is included to contrast the patterns and pinpoint the cause of read count bias. Lastly, it is shown that the sample-permuting gene-set enrichment analysis (GSEA) [21] is highly affected by the read count bias and hence generates a considerable number of false positives, while the preranked GSEA does not generate false positives by the read count bias. See also the paper by Zheng and colleagues for other types of biases in quantifying RNA-seq gene expression rather than in DE analysis [22]. We also note a recent study reporting that small dispersions result in high statistical power in DE analysis of RNA-seq data [23].

## Results and Discussion

### The read count bias is pronounced with technical replicates, but is rarely observed with unrelated replicates

In DE analysis of RNA-seq count data between different sample groups, it is known that genes with a larger read count (or longer genes) are more likely to be differentially expressed [15, 16]. We tested such a pattern by plotting a gene differential score (SNR: signal to noise ratio) for four RNA-seq read count datasets denoted as Marioni, MAQC-2, TCGA KIRC and TCGA BRCA, respectively with each having two sample groups. See Table 1 and Supplementary Material (Additional file 1) for the detailed information of each dataset. The SNR for gene *g*_*i*_ is defined as follows:

$$ S N{R}_i = \frac{\mu_{i1}-{\mu}_{i2}}{\sigma_{i1}+{\sigma}_{i2}} $$

where *μ*_*ik*_ and *σ*_*ik*_ are the mean and standard deviation of *i*th gene *g*_*i*_ and sample group *k* (*k* = 1 or 2) for the read count data normalized with the DESeq median method [13]. Although the variances of the normalized counts in each gene may not be identical if the depths of each sample are different, they share the same quadratic term in the *negative binomial* variance across the samples. In other words, SNR score can largely represent the distribution of gene differential expression score (effect size/standard error). Thus, these normalized counts have been used for GSEA of RNA-seq data [24–26].

**Fig. 1:**
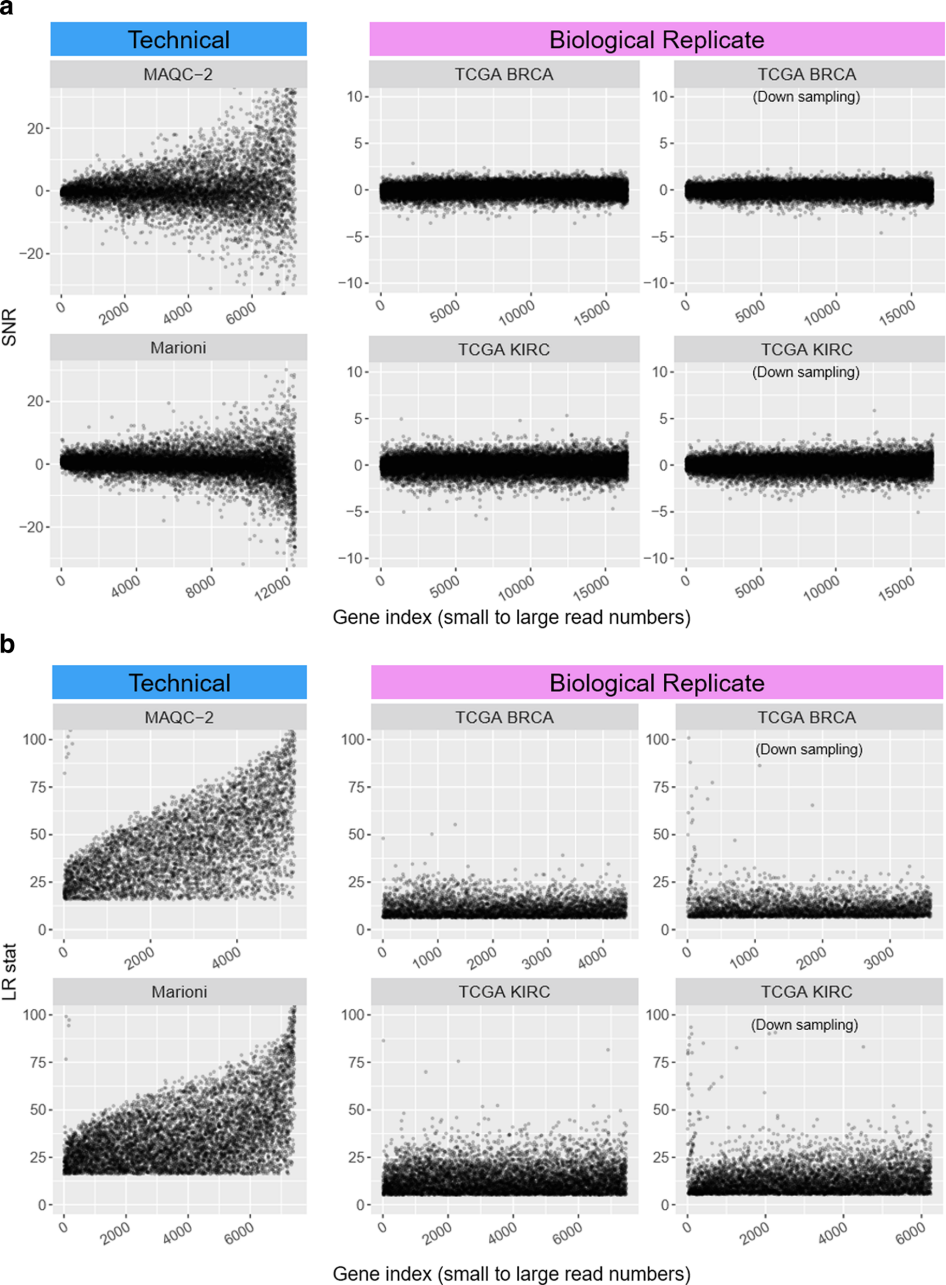
**a** Distributions of signal-to-noise ratio (SNR) against read count. Read count bias was compared between two technical (MAQC-2 and Marioni dataset) and two unrelated (TCGA BRCA and KIRC dataset) replicate datasets. For a fair comparison regarding the replicate number and sequencing depth, TCGA BRCA and KIRC data were down-sampled and down-replicated to the Marioni dataset level (third column figures) from the original datasets (second column figures). **b** The likelihood ratio test statistic instead of the SNR was also plotted only for the significant genes

The SNR scores for the four datasets were plotted in the ascending order of the mean read count of each gene in Fig. 1 (a). The ‘read count bias’ was well represented with the two datasets (Marioni and MAQC-2) where genes with a larger read count had more scattered distributions of the gene scores. This pattern indicates that genes with a larger read count are more likely to have a higher level of differential scores. Curiously, many of the read count data from TCGA [27] did not show such a bias but exhibited an even SNR distribution.

A possible reason for the two distinctly different SNR patterns was the sample replicate type: The former two (Marioni and MAQC-2 dataset) were composed of technical replicate samples while the latter two (TCGA KIRC and TCGA BRCA) of biological replicates obtained from different patient samples. Besides, the replicate size and sequencing depth may affect the power of DE analysis. Because the replicate numbers are equally set to be seven for all the four datasets, we examined the effect of the sequencing depth by down-sampling the counts. The read counts in the two TCGA datasets were down-sampled to the Marioni dataset level which had the lowest depth among the four: We computationally down-sampled the data using binomial distribution [28] because TCGA provided only the level-three count data. Then, the SNR scores for the two TCGA datasets were plotted again. Interestingly, the SNR scores for the down-sampled TCGA datasets still exhibited nearly even SNR distributions except for some small read counts (Fig. 1a). This preliminary test suggests that the *sample replicate type* (more precisely, the gene dispersion which will be described in the next section) is a key factor that determines the read count bias, whereas the replicate number and the depth exercise only a limited effect. To corroborate the evidence, we analyzed probability models and conducted a simulation test in the following sections.

The SNR scores are also depicted for the voom (TMM)-transformed data [29] which exhibited similar patterns except for the unexpected large variations with some small counts in the technical replicate data (Additional file 2: Figure S1). Because the SNR does not explicitly identify the DE genes, the likelihood ratio test (dubbed *naïve LRT*) statistic for the significance cutoffs (Marioni, MAQC-2: FDR < 0.0001; TCGA KIRC, TCGA BRCA: FDR < 0.05) was also plotted in Fig. 1 (b) using the glm.nb() function in the MASS R package instead of the SNR scores. See Supplementary Material (Additional file 1) for the implementation of the naïve LRT method. The LRT statistic demonstrated similar bias patterns as the SNR.

### Modeling the read count data and comparison of the gene dispersion distributions between different replicate types

The main difference between technical and unrelated replicates is the gene-wise variance across the samples. The technical replicate data are generated from the same samples, so most of its variation comes from the experimental noise such as random sampling. In such a case, the read count of *i*th gene in *j*th sample, denoted by *X*_*ij*_, can be simply assumed to have a Poisson distribution *X*_*ij*_ ~ *Poisson*(*μ*_*ij*_) where the mean and variance are the same as *μ*_*ij*_ [9]. However, unrelated replicates also involve biological variations between individuals [13, 30]. In such a case, the read count *X*_*ij*_ is modelled by a negative binomial (NB) distribution to account for the increased variability, and denoted as *X*_*ij*_ ~ *NB*(*μ*_*ij*_, *σ*_*ij*_^2^) where *μ*_*ij*_ and *σ*_*ij*_^2^ are the mean and variance, respectively. Its variance is given as *σ*_*ij*_^2^ = *μ*_*ij*_ + *α*_*i*_ *μ*_*ij*_^2^, where *α*_*i*_ is the *dispersion* coefficient for *g*_*i*_ that determines the amount of additional variability [14]. In particular, the NB distribution becomes a Poisson distribution when *α*_*i*_ approaches 0.

**Fig. 2:**
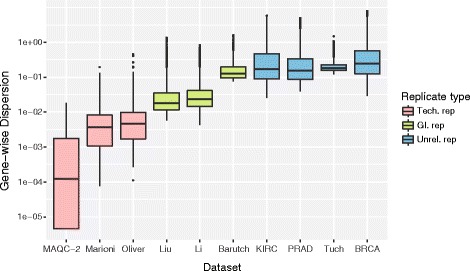
Distributions of gene dispersions (log scale) for ten published RNA-seq datasets. Three technical (*pink*), three GI (*green*) and four unrelated (*blue*) replicate datasets were analyzed. Dispersions were estimated using the edgeR package

The dispersion coefficient *α*_*i*_ for each gene can be estimated using the edgeR package [14] and the distribution of the estimated *α*_*i*_’s for ten publicly available RNA-seq count datasets are shown in Fig. 2. The first three are technical replicates and their median dispersions ranged between 0.00013 and 0.0046. The last four datasets were of unrelated replicates whose median dispersions ranged between 0.15 and 0.28. The middle three datasets (fourth to sixth) were generated from cell lines and represent identical genetic backgrounds (GI replicates). These cell line data exhibited an intermediate range of dispersions between those of technical and unrelated replicates (0.018 ~ 0.127). Among them, the GI and unrelated replicates can be called biological replicates. See the reference [31] for a similar classification of the replicate types. Of note, most gene dispersions in unrelated replicate datasets were larger than 0.1 (blue boxes). The dispersion values estimated using the naïve LRT were also plotted (Additional file 2: Figure S2). They exhibited similar distributions as in Fig. 2 but with overall higher variations. This difference may be ascribed to the tight shrinkage-based dispersion estimation in the edgeR method.

### Gene dispersion is the key determinant of the read count bias: simulation tests

**Fig. 3:**
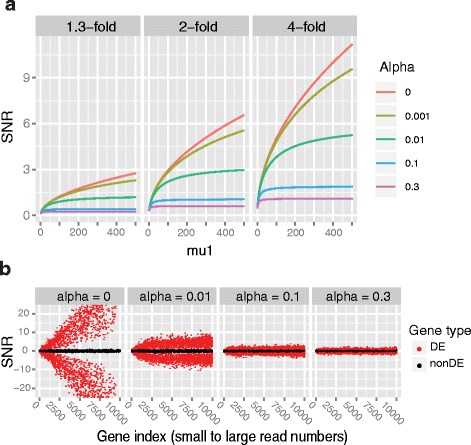
Effect of gene dispersion on the read count bias. **a** For a given fold-change (*f* = 1.3, 2, 4-fold) and a dispersion value (alpha = 0, 0.001, 0.01, 0.1 and 0.3), SNR for each read count (*μ*_1_) was depicted based on the equation (1). **b** SNR distributions of simulated genes for different dispersion values (alpha). Mean read counts were sampled from a high depth dataset (TCGA KIRC)

The SNR score for biological replicate data is represented as

1 $$ S N{R}_i = \frac{\mu_{i1}-{\mu}_{i2}}{\sigma_{i1}+{\sigma}_{i2}}=\frac{\mu_{i1}-{\mu}_{i2}}{\sqrt{\mu_{i1}+{\alpha}_i{\mu}_{i1}^2}+\sqrt{\mu_{i2}+{\alpha}_i{\mu}_{i2}^2}}, $$

where *μ*_*ik*_ and *σ*_*ik*_ are the mean and standard deviation of the normalized counts for *i*th gene in the sample group *k* = 1 or 2. For the technical replicate case where the dispersion coefficient α_*i*_ is close to 0, the SNR value is approximated to,

$$ S N{R}_i \approx \frac{\mu_{i1}-{\mu}_{i2}}{\sqrt{\mu_{i1}}+\sqrt{\mu_{i2}}}=\sqrt{\mu_{i1}}-\sqrt{\mu_{i2}} $$

which directly depends on the read counts. This accounts for the increasing SNR variation with the technical replicate data in Fig. 1. However, for biological replicate data where α_*i*_ is not negligible in (1) and the SNR is estimated as

2 $$ \left| SN{R}_i\right|=\left|\frac{1-1/ f}{\sqrt{1/{\mu}_{i1}+{\alpha}_i}+\sqrt{1/\left({\mu}_{i1} f\right)+{\alpha}_i/{f}^2}}\right|\le \left(\frac{1-1/ f}{1+1/ f}\right)\cdot \left|\frac{1}{\sqrt{1/{\mu}_{i1}+{\alpha}_i}}\right|\le \min \left(\frac{1}{\sqrt{\alpha_i}},\sqrt{\mu_{i1}}\right) $$

using the inequality 1/(*μ*_*i*1_ *f*) ≥ 1/(*μ*_*i*1_ *f* ^2^) where *f* = *μ*_*i*1_/*μ*_*i*2_ is the fold change value (We assume *μ*_*i*1_ ≥ *μ*_*i*2_ without loss of generality). Similarly, the lower bound is obtained using inequality *α*_*i*_/*f* ^2^ ≤ *α*_*i*_/*f* as

3 $$ \left| SN{R}_i\right|\ge \left(1-1/\sqrt{f}\right)\cdot \left|\frac{1}{\sqrt{1/{\mu}_{i1}+{\alpha}_i}}\right|\ge c(f)\cdot \max \left(\frac{1}{\sqrt{\alpha_i}},\sqrt{\mu_{i1}}\right) $$

where $c(f)=\frac{1}{\sqrt{2}}\cdot \left(1-\frac{1}{\sqrt{f}}\right)$. The ratio of the coefficients of the two bounds in (2) and (3) was also tightly bounded as $1<\left(\frac{1-1/ f}{1+1/ f}\right)/\left(1-1/\sqrt{f}\right)<1.21$ for any fold-change *f*. The upper bound (2) indicates the SNR values for biological replicate data are *bounded* by a constant $1/\sqrt{\alpha_i}$ irrespective of the mean read count and the fold change level. The relationship between SNR and read count (*μ*_*i*1_) is demonstrated in Fig. 3a for different fold change (*f*) and dispersion values. For a dispersion value of 0.1 or higher, the SNR exhibited nearly a ‘flat’ distribution except for some small read counts (< tens), while the SNR rapidly increased for smaller dispersion values. This pattern was observed across different levels of the fold change values. This result accounts for both the ‘divergent’ SNR distribution with the technical replicates and the ‘even’ SNR distribution with the unrelated replicates shown in Fig. 1.

Note that the |*SNR*_*i*_| value in (2) is also bounded by $\sqrt{\mu_{i1}}$, which implies if the read count is sufficiently small, the SNR exhibits a read count bias. This accounts for the ‘local’ read count bias at small read counts (< tens) for large dispersions (>0.1) in Fig. 3a. Therefore, if the dispersion value increases, the region for the local read count bias is reduced. Similarly, if sufficiently large sequencing depth is used, the curves in Fig. 3a starts from some large read count, and the read count biases will be rather alleviated. An inference with two-sample *T*-statistic results in similar relationships between dispersion, read count, fold change as well as replicate size (Additional file 1: Supplementary Material).

Based on this reasoning, we simulated the read count data to show how the SNR scores are distributed for each replicate model (see Methods). Read count data for 10,000 genes were simulated using Poisson or negative binomial distributions for four different dispersion values 0, 0.01, 0.1 and 0.3. The means of the 10,000 genes were randomly sampled from the TCGA KIRC RNA-seq data. Therefore, this simulation compares the SNR distributions of the technical (α ≤ 0.01) and unrelated replicate (α ≥ 0.1) data at the same ‘high depth’ of a TCGA dataset. Among the genes, 30% of the genes were chosen and the mean of their test group counts were increased or decreased by 1.3 ~ 4-folds to generate the DE genes (see Methods). Then, the SNR values for each dispersion value were depicted in Fig. 3b, which reproduced the SNR patterns for the real count datasets (Fig. 1). For data with zero or a small dispersion (≤0.01), which corresponds to the technical or some GI replicates, the SNR scores of DE genes (red dots) were more scattered as their read counts were increased. However, for data with 0.1 or higher dispersion, the SNR variation became nearly independent of the read counts. Then, the same experiment was performed at the low depth of Marioni. In other words, the mean of 10,000 genes were sampled from the Marioni data, which resulted in similar SNR patterns (data not shown). This indicates the Poisson-like small variance in the technical replicate data is the primary cause of the read count bias which cannot be removed by simply increasing the sequencing depth.

The gene length bias [15] can similarly be explained using gene dispersion. If *μ*_*i*1_ is represented as c*N*_*i*_ *L*_*i*_ where c is a proportionality constant, *N*_*i*_ is the total number of transcripts and *L*_*i*_ is the length of gene *i*, it can be easily shown that the *SNR*_*i*_ in (1) is also bounded by the same constant $1/\sqrt{\alpha}$ whatever the gene length *L*_*i*_ is, while the *SNR*_*i*_ becomes proportional to $\sqrt{L_i}$ under the Poisson model. This means that the gene length bias also disappears with some large dispersion values.

### Gene dispersion is the key determinant of the read count bias: RNA-seq data analysis

The down-sampling analysis in a previous Section is useful for prioritizing the key factor for the read count bias. However, the Marioni data were generated at quite a low depth with a specific purpose of comparing RNA-seq with microarray, and hence the influence of genes with low counts can be amplified. The key point of this paper is that the well-known read count bias (and gene length bias) nearly dissipates in many (or most) unrelated replicate data with a commonly used depth (more than hundreds of median read count) and the small dispersion is the primary cause of the read count bias.

**Fig. 4:**
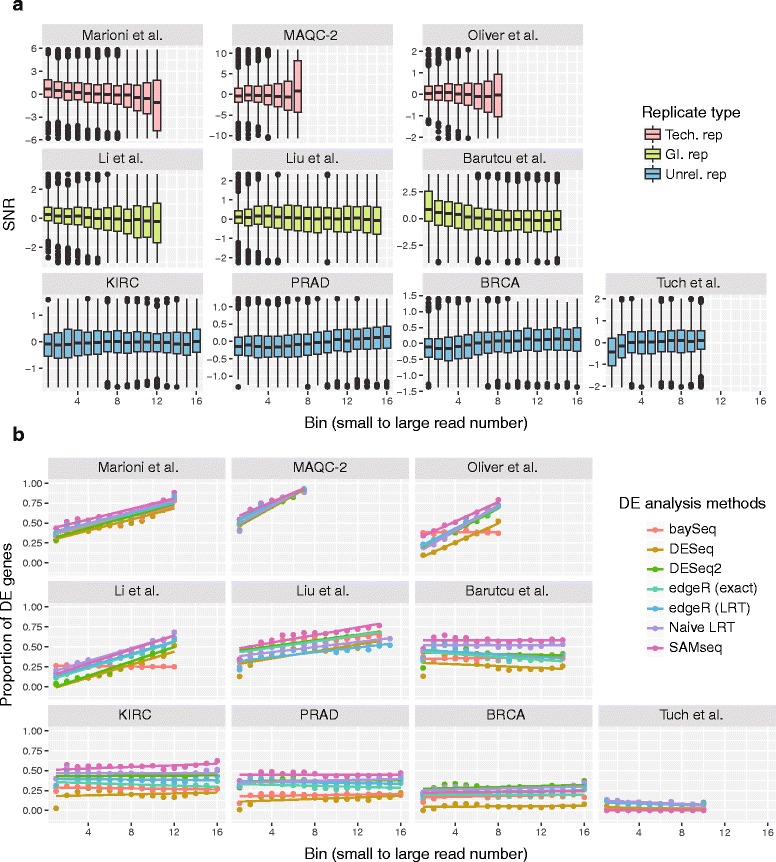
Comparison of read count bias for three different replicate type datasets. For ten published RNA-seq datasets, **a** the boxplots of SNRs are depicted against the read counts. Each bin contains 1000 genes. Each row of figures represents technical (MAQC-2, Marioni, Oliver dataset; *pink*), GI (Liu, Li and Barutcu dataset; *green*), and unrelated (TCGA BRCA, KIRC, PRAD and Tuch dataset; *blue*) replicate data, respectively. **b** The proportions of DE genes in each bin were plotted for each dataset. The DE genes were obtained by using the R packages baySeq, DESeq, DESeq2, edgeR, MASS (naïve LRT) and SAMseq

To demonstrate this, the SNR distributions of ten publicly available RNA-seq read count datasets were depicted (as boxplots) in Fig. 4a in their original depths. See Table 1 and Supplementary Material for a detailed description of the RNA-seq datasets. Among them, only the seven samples in each condition (as used for Fig. 1) were used for the TCGA KIRC and TCGA BRCA data. Using the full dataset resulted in too many DE genes to analyze the bias pattern. For example, using baySeq for the full dataset (FDR < 0.05), nearly 100% genes were DE genes. All the four unrelated replicate datasets exhibited nearly even SNR distributions (except for the first bin for some datasets) while the three technical replicate data exhibited a clear read count bias. The three GI replicate datasets split in their patterns depending on their dispersion distributions. The Barutcu data [32] which compared the gene expression between MCF7 and MCF10A cell lines had dispersion values as large as those of unrelated replicate datasets and demonstrated an even SNR distribution, while the other two cell line data, Liu (MCF7 vs E2-treated MCF7) and Li (LNCaP vs. androgen-treated LNCaP) data [33, 34] had smaller dispersion values (Fig. 2) and exhibited a moderate read count bias.

**Table 1:** The 16 public RNA-seq data tested

Name	Experiment	Test group size	Control group size	Replicate type
Marioni [9]	Human liver vs. kidney	7	7	Technical
MAQC-2 [41]	HBRR vs. SUHRR	7	7	Technical
Oliver [42]	Head tissue of male vs. female Drosophila melanogaster	10	10	Technical
Barutcu [32]	MCF7 vs. MCF10A	3	3	GI
Liu [33]	10nM E2-treated vs. control MCF7	7	7	GI
Li [34]	Androgen-treated vs. control LNCaP cell line	4	3	GI
TCGA KIRC [27]	Human renal clear cell carcinoma vs. matched normal tissue	7	7	Unrelated
TCGA BRCA [43]	Human invasive breast cancer vs. matched normal tissue	7	7	Unrelated
TCGA PRAD [43]	Human prostate adenocarcinoma vs. matched normal tissue	15	15	Unrelated
Tuch [44]	Human oral squamous cell carcinoma vs. matched normal tissue	3	3	Unrelated
ModencodeFly [37]	L1 Larvae vs. Embryos (12–14 h)	4	5	Technical
	White pre-pupae (12 h) vs. L1 Larvae	5	4	Technical
	Adult male (1 day) vs. White pre-pupae (12 h)	5	5	Technical
	Pooled Larvae vs. pooled embryos (12–24 h)	6	6	Unrelated
	Pooled pupae vs. pooled larvae	6	6	Unrelated
	Pooled adult male vs. pooled pupae	3	6	Unrelated

*Abbreviation*: *GI* genetically identical, *HBRR* Ambion First Choice Human Brain Reference RNA, *SUHRR* Stratagene Universal Human Reference RNA

Then, the DE gene distributions along the read count were analyzed using seven different DE analysis methods and corresponding R packages which are available from the Bioconductor (DESeq [24], edgeR [31], baySeq [35], SAMseq [28], DESeq2 [36]) (https://www.bioconductor.org) and CRAN (MASS) (https://cran.r-project.org). The proportions of DE genes in each bin of 1000 genes for each method were depicted in Fig. 4b. A significance criterion FDR < 0.0001 was used for Marioni, MAQC-2 and Liu data where a great number of DE genes were detected and the criterion FDR < 0.05 was used for other datasets. In all the technical replicates and two GI replicates (Liu and Li), the proportion of DE genes increased as the read count was increased for most of the DE analysis methods. On the contrary, the proportion of DE genes was largely independent of the read count for all the unrelated replicate datasets and one GI dataset (Barutcu). Therefore, the read count bias can be largely predicted from the replicate type in many cases. However, for GI replicate case, it is worth checking the dispersion or the SNR distribution prior to the DE analysis. Unrelated replicate data with very small dispersion values, if any, can also have a read count bias and can be warned in advance.

In addition, we analyzed the fly developmental transcriptome data [37] that contained both technical and biological replicate data for four different developmental stages, and very similar results were obtained. See Figure S3 and S4 (Additional file 2).

### Small gene dispersions in read count data result in false positives in the sample-permuting gene-set enrichment analysis

**Fig. 5:**
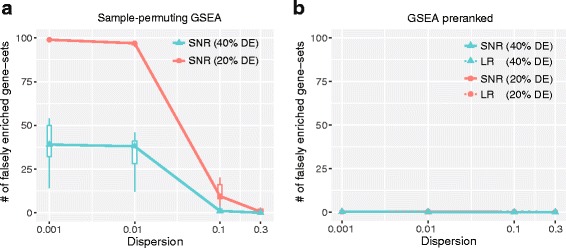
The effect of gene dispersion on GSEA. **a** The sample-permuting GSEA results in a great number of false positives for small dispersion values. **b** The preranked GSEA resulted in no false positives for all the dispersion values

Because the effect of read count bias on GO analysis has been explored earlier [16], we investigate its effect on GSEA [21] for different dispersion values. To this end, read counts for 10,000 genes and 20 samples including ten case and ten control samples were simulated using NB distribution for four different levels of dispersion values (0.001, 0.01 and 0.1, and 0.3) as described in Methods. These genes were then categorized into 100 non-overlapping gene-sets. Among the 10,000 genes, α % (α =10, 20, 30 or 40) of the total genes were randomly selected and set to be DE genes (half up, half down, two-fold change). These simulated datasets were normalized using DESeq median method [13] and the conventional sample-permuting GSEA with the SNR gene score was applied for the normalized count data using the GSEA-R code [21]. This test was repeated ten times and the average number of significant (FDR < 0.05) gene-sets were depicted in Fig. 5. Because the DE genes were randomly selected, no gene-set was expected to be ‘enriched’ with the DE genes. (Thus, ‘significant’ gene-set obtained here is either referred to as ‘falsely enriched’ or ‘false positive’ gene-set). However, the analysis of data with small dispersion values (≤0.01) exhibited a great number of significant gene-sets. For 10, 20 and 30% DE genes, the false positives rate was similar to each other, but was overall reduced for 40% DE genes. Recall that for small dispersion values, the read counts heavily affected the SNR scores of DE genes (Fig. 3). In other words, only a few DE genes with a large read count can greatly affect the gene-set score. The number of falsely enriched gene-sets rapidly decreased as the dispersion was increased, and only a few or no gene sets were significant for the large dispersion value of 0.3. This result indicates that the small gene dispersions observed in technical or some of the GI replicates can considerably inflate the gene-set scores and result in a great number of false positive gene-sets. Such false positives cannot be removed even by the sample-permutation procedure of GSEA.

Then, the same simulation datasets were analyzed using the preranked GSEA which only makes use of the gene ranks to test the gene-sets. Interestingly, no false positives were detected for all the dispersion values and gene scores. So, the preranked GSEA is recommendable for controlling the false positives caused by the read count bias. This gene-permuting method, however, is likely to result in false positives caused by the inter-gene correlations which is not simulated in this study [26, 38]. Thus, a further study is required to find the method that exhibits better overall false positive control taking into account both the read count bias and the inter-gene correlation.

## Conclusion

Previous studies have reported a bias in differential analysis of RNA-seq count data regarding gene length (or read count) and its effect on GO analysis [15, 16]. However, it has been observed that such a bias is not always present [19, 20]. In this study, it is shown that the gene dispersion is the key factor that causes the read count bias (and gene length bias) and the sequencing depth and replicate size also had some effects on the bias for small read counts. To this end, mathematical inferencing, model-based simulation and tests with 16 RNA-seq datasets were performed. Then, it is shown that the read count bias is mostly confined to technical replicate or some of the genetically identical replicate data which have small dispersion values. On the other hand, biological replicates composed of unrelated samples had much larger dispersion values, which mostly removed the read count bias except for very small counts. Thus, for the extremely small counts such as the single cell data, we expect some read count bias. However, this topic may require further research because somewhat different (more generalized) variance model may be required for the single cell data, and the DE analysis methods used for the ‘bulk’ RNA-seq data may not perform best with the single cell data [39, 40]. Lastly, it was shown that the small dispersions cause a considerable number of false positives in the sample-permuting GSEA method, whereas large dispersions resulted in only a few. However, the preranked GSEA did not result in false positives at all from the read count bias.

Overall, this study recommends using unrelated replicates for RNA-seq differential expression analysis and warns of read count bias for some of the genetically identical replicates for which an appropriate adaptation algorithm or the preranked GSEA may be applied for an unbiased functional analysis [16, 20].

## Methods

### Simulation of read count data

The read count *X*_*ij*_ of gene *i* and sample *j* was generated using Poisson or negative binomial distribution depending on the gene dispersion of each simulation dataset

$$ {X}_{ij}\sim Poisson\left({\mu}_{ij}\right)\kern2.5em \mathrm{f}\mathrm{o}\mathrm{r}\ \mathrm{dispersion}=0 $$

$$ {X}_{ij}\sim N B\left({\mu}_{ij},{\sigma}_{ij}^2\right)\kern3em \mathrm{f}\mathrm{o}\mathrm{r}\ \mathrm{dispersion}=0.01,\ 0.1\ \mathrm{o}\mathrm{r}\ 0.3 $$

where *μ*_*ij*_ is the mean and *σ*_*ij*_^2^ is the variance. Each simulated dataset contained 10000 genes and 20 samples (ten samples for each group). The mean read counts for simulated genes were determined by randomly selecting 10000 median gene counts from TCGA KIRC (Fig. 3b). To generate DE genes, a random number between 1.3 ~ 4 was either multiplied or divided to the gene’s mean for 3000 randomly chosen genes (30%). Then, using rpois and rnbinom R functions, the read counts for technical and biological replicate data were simulated, respectively. The reciprocal of dispersion value was used for the ‘size’ option in rnbinom function.

## Additional files

Additional file 1: Inference for two-sample *T*-statistic, Naïve LRT implementation, Publicly available RNA-seq datasets tested in this study. (DOCX 41 kb)

Additional file 2: Figure S1-S4. (DOCX 649 kb)

## Abbreviations

GI replicate: Genetically identical replicate
LRT: Likelihood ratio test
NB: Negative binomial
SNR: Signal to noise ratio
