# Supplementary material for: Gene dispersion is the key determinant of the read count bias in differential expression analysis of RNA-seq data
Source: BMC Genomics. 2017 May 25;18:408. doi: 10.1186/s12864-017-3809-0 (PMC5445461; doi:10.1186/s12864-017-3809-0)
Supplement: Supplementary file 1 — Inference for two-sample T-statistic, Naïve LRT implementation, Publicly available RNA-seq datasets tested in this study. (DOCX 41 kb) [file 12864_2017_3809_MOESM1_ESM.docx]

Supplementary Material

**Inference for two-sample *T*-statistic**

Although we may not assume the read counts have a normal distribution, analysis of two-sample *t*-statistic may provide a useful insight on the read count bias. For simplicity, the replicate sizes in the test and control conditions are the same as *n* and only one gene is considered. Suppose $(X_{1},X_{2}\ldots,X_{n})$ and $(Y_{1},Y_{2}\ldots,Y_{n})$ are the read counts in test and control conditions, respectively, where $X_{i}\sim NB(\mu_{1},\sigma_{1}^{2}$) and $Y_{i}\sim NB(\mu_{2},\sigma_{2}^{2}$). Then, for $\mu_{1}=\bar{X}$ and $\mu_{2}=\bar{Y}$, the two sample T-statistic is given as

$T=\frac{\sqrt{n} (\mu_{1}-\mu_{2})}{\sqrt{{(\mu}_{1}+\alpha\mu_{1}^{2})+{(\mu}_{2}+\alpha\mu_{2}^{2})}}=\frac{\sqrt{n} (f-1)}{\sqrt{{\alpha\left( f^{2}+1 \right)+(f+1)/\mu}_{2}}}\leq\sqrt{n}\cdot min(\frac{c_{1}}{\sqrt{\alpha}}, c_{2}\sqrt{\mu_{2}})$,

where $f=\frac{\mu_{1}}{\mu_{2}}\geq1$, $c_{1}=\frac{f-1}{\sqrt{f^{2}+1}}$ and $c_{2}=\frac{f-1}{\sqrt{f+1}}$. So, if $\alpha$ is sufficiently large such that ${\alpha\left( f^{2}+1 \right)\geq(f+1)/\mu}_{2}$, T is bounded by $\frac{c_{1}\sqrt{n}}{\sqrt{\alpha}}$ which is independent of the read count. Otherwise, it is bounded by $c_{2}\sqrt{n}\sqrt{\mu_{2}}$ which implies the ‘local’ read count bias at small read counts. Compared to SNR score (2), *T*-statistic is also affected by the square-root of replicate size.

**Naïve LRT implementation**

According to McCarthy et al. [1], the number of read counts in sample *i* that mapped to gene *g* ($\mu_{gi}$) can be modeled by following log-linear model.

$$\log\mu_{gi}=\mathbf{X}_{i}^{T}\beta_{g}+\log N_{i}$$

where $\mathbf{X}_{i}^{T}$ is a vector of covariates that represents the experimental condition of RNA-seq sample *i*, $\beta_{g}$ is the regression coefficient of gene *g*, and $N_{i}$ is the total number of mapped reads in sample *i*.

To estimate the effect of each gene to the phenotype, we performed log-ratio test comparing two models, with or without covariate term. It was done by using glm.nb() function in the MASS R package. An example code for calculating the LRT statistic of a gene (ENSG00000000003 in MAQC dataset) is in the following.

| library(MASS)  countTable = read.delim('http://bowtie-bio.sourceforge.net/recount/countTables/maqc_count_table.txt', row.names=1)  countTable = data.matrix(countTable) # MAQC-2 read count table.  $count<-countTable[1,]$ # ENSG00000000003 is in the first row of countTable  $class<-rep\left( c\left( 0,1 \right), each=7 \right)$# sample class  $totalCount<-log2(colSums(countTable))$ #log2 scale of total read count of each sample.  $model1<-glm.nb(count\sim class+totalCount)$ # model with covariate term  $model2<-update(model1, .\sim. -class)$ # model without covariate term.  $anov<-anova(model1, model2)$ # Comparison of two models  $LR<-anov\$`LR stat.`[2]$ # Log-ratio test statistics |
| --- |

Then, the LR statistic is evaluated as 30.633. Also, dispersion coefficient can be obtained as follows:

| $dispersion<-1/model1\$theta$ # 0.026 |
| --- |

**Publicly available RNA-seq datasets tested in this study**

We used sixteen publicly available RNA-seq read count datasets including three technical replicate datasets (Marioni, MAQC-2, and Oliver), three genetically identical (GI) replicate datasets (Barutcu, Liu and Li) and four unrelated replicate datasets (TCGA KIRC, BRCA, PRAD and Tuch). For each dataset, genes having less than five median read counts were excluded from the analysis.

- Marioni dataset: ~20 million reads mapped (median: 44 reads). Total mRNAs extracted from human liver and kidney sample were sequenced on 14 lanes of Illumina GA 1 (seven lanes each). In the early stage of RNA-seq study, Marioni and colleagues produced this dataset to estimate the technical variance of Illumina sequencing and compared the capability of identifying DE genes with that of the Affymetrix array. A processed RNA-seq read count matrix was downloaded from the supplemental table 2 of the Marioni and colleagues’ paper [2] .
- MAQC-2 dataset: ~18 million read mapped (median: 51 reads). The MicroArray Quality Control Project (MAQC) measured the gene expression levels from two distinct RNA samples (Ambion’s human brain reference RNA and Stratagene’s human universal reference RNA) on four titration pools on seven microarray platforms [3]. MAQC-2 is a part of MAQC project. Bullard and colleagues [4] sequenced the two RNA samples on Illumina Genome Analyser II and then, evaluated the statistical methods for normalization and differential expression. The MAQC-2 count data and phenotype information were downloaded from the ReCount [5] web page (http://bowtie-bio.sourceforge.net/recount).
- Oliver dataset: ~32 million reads mapped (median: 52 reads). The mRNA from male and female head tissue of Drosophila melanogaster to identify sex-specfic alternative splicing events. Submission number of Sequence Read Archive is SRA012403 [6]
- Barutcu dataset: ~91 million reads mapped (median: 287 reads). The gene expression between MCF10A and MCF7 was compared and each had three GI replicates. The GEO accession number is GSE71862 [7]
- Liu dataset: ~358 million reads mapped (median: 558 reads). The gene expression between 10nM E2-treated and control MCF7 was compared. Each has seven GI replicates and each replicate was sequenced at multiple sequencing depths (2.5 ~ 30 million) and we used samples of 30 million reads. The GEO accession number is GSE51403 [8].
- Li dataset: ~9 million reads mapped (median: 34 reads). Gene expression between androgen-treated and control LNCaP cell line was measured. Each condition has three and four replicates respectively. RNA-seq raw count table was downloaded as ‘pnas expression.txt’ from https://sites.google.com/site/davismcc/useful-documents/ [9].
- TCGA KIRC vs normal dataset: The RNA-seq count data of renal clear cell carcinoma (KIRC) patients’ tumour and matched normal samples were obtained from The Cancer Genome Atlas (TCGA) data portal [10, 11]. Due to the large size, we used a subset composed of randomly selected seven paired (cancer/matched normal) samples. ~761 million reads mapped in this subset (median: 1035 reads).
- TCGA BRCA dataset: The RNA-seq count data of the invasive breast cancer patients’ tumour and matched normal samples were downloaded from the TCGA Data portal [12]. Similar to KIRC dataset, a subset composed of randomly selected seven paired (cancer/matched normal) samples were used. ~692 million reads mapped in subset (median: 973 reads).
- TCGA PRAD dataset: The RNA-seq count data of prostate adenocarcinoma patient’s tumour and matched normal samples were downloaded from the TCGA Data portal. Each had 15 replicates. ~1.7 billion reads mapped (median: 1062 reads) [12]
- Tuch dataset: ~83 million reads mapped (median: 359 reads). Gene expression between oral squamous cell carcinoma and its matched normal samples were compared. Each condition has three unrelated replicates. RNA-seq count data is available in Table S1 of the paper [13]
- ModencodeFly dataset: It contains read counts of 30 distinct developmental stages (12 embryonic, 6 larval, 6 pupal, 3 male and 3 female adult stage) of Drosophila melanogaster [14]. Re-Count database provides its original (composed of the technical replicates of each developmental stage, 147 samples) and pooled (30 samples) RNA-seq data [5]. ~ 2.3 billion reads were mapped (median: 263 in the original and 1213 in the pooled data).

1. McCarthy DJ, Chen Y, Smyth GK: **Differential expression analysis of multifactor RNA-Seq experiments with respect to biological variation**. *Nucleic Acids Res* 2012, **40**(10):4288-4297.

2. Marioni JC, Mason CE, Mane SM, Stephens M, Gilad Y: **RNA-seq: An assessment of technical reproducibility and comparison with gene expression arrays**. *Genome Research* 2008, **18**(9):1509-1517.

3. Shi LM, Reid LH, Jones WD, Shippy R, Warrington JA, Baker SC, Collins PJ, de Longueville F, Kawasaki ES, Lee KY *et al*: **The MicroArray Quality Control (MAQC) project shows inter- and intraplatform reproducibility of gene expression measurements**. *Nature Biotechnology* 2006, **24**(9):1151-1161.

4. Bullard JH, Purdom E, Hansen KD, Dudoit S: **Evaluation of statistical methods for normalization and differential expression in mRNA-Seq experiments**. *Bmc Bioinformatics* 2010, **11**.

5. Frazee AC, Langmead B, Leek JT: **ReCount: A multi-experiment resource of analysis-ready RNA-seq gene count datasets**. *Bmc Bioinformatics* 2011, **12**.

6. Malone JH, Oliver B: **Microarrays, deep sequencing and the true measure of the transcriptome**. *BMC Biol* 2011, **9**:34.

7. Barutcu AR, Lajoie BR, McCord RP, Tye CE, Hong D, Messier TL, Browne G, van Wijnen AJ, Lian JB, Stein JL *et al*: **Chromatin interaction analysis reveals changes in small chromosome and telomere clustering between epithelial and breast cancer cells**. *Genome Biol* 2015, **16**(1):214.

8. Liu YW, Zhou J, White KP: **RNA-seq differential expression studies: more sequence or more replication?** *Bioinformatics (Oxford, England)* 2014, **30**(3):301-304.

9. Li H, Lovci MT, Kwon YS, Rosenfeld MG, Fu XD, Yeo GW: **Determination of tag density required for digital transcriptome analysis: application to an androgen-sensitive prostate cancer model**. *Proc Natl Acad Sci U S A* 2008, **105**(51):20179-20184.

10. Benidt S, Nettleton D: **SimSeq: a nonparametric approach to simulation of RNA-sequence datasets**. *Bioinformatics* 2015.

11. Cancer Genome Atlas Research N: **Comprehensive molecular characterization of clear cell renal cell carcinoma**. *Nature* 2013, **499**(7456):43-49.

12. Cancer Genome Atlas Research N, Weinstein JN, Collisson EA, Mills GB, Shaw KR, Ozenberger BA, Ellrott K, Shmulevich I, Sander C, Stuart JM: **The Cancer Genome Atlas Pan-Cancer analysis project**. *Nat Genet* 2013, **45**(10):1113-1120.

13. Tuch BB, Laborde RR, Xu X, Gu J, Chung CB, Monighetti CK, Stanley SJ, Olsen KD, Kasperbauer JL, Moore EJ *et al*: **Tumor transcriptome sequencing reveals allelic expression imbalances associated with copy number alterations**. *PLoS One* 2010, **5**(2):e9317.

14. Graveley BR, Brooks AN, Carlson J, Duff MO, Landolin JM, Yang L, Artieri CG, van Baren MJ, Boley N, Booth BW *et al*: **The developmental transcriptome of Drosophila melanogaster**. *Nature* 2011, **471**(7339):473-479.
