# Supplementary material for: Gene dispersion is the key determinant of the read count bias in differential expression analysis of RNA-seq data
Source: BMC Genomics. 2017 May 25;18:408. doi: 10.1186/s12864-017-3809-0 (PMC5445461; doi:10.1186/s12864-017-3809-0)
Supplement: Supplementary file 2 — (DOCX 649 kb) [file 12864_2017_3809_MOESM2_ESM.docx]

Supplementary Figures


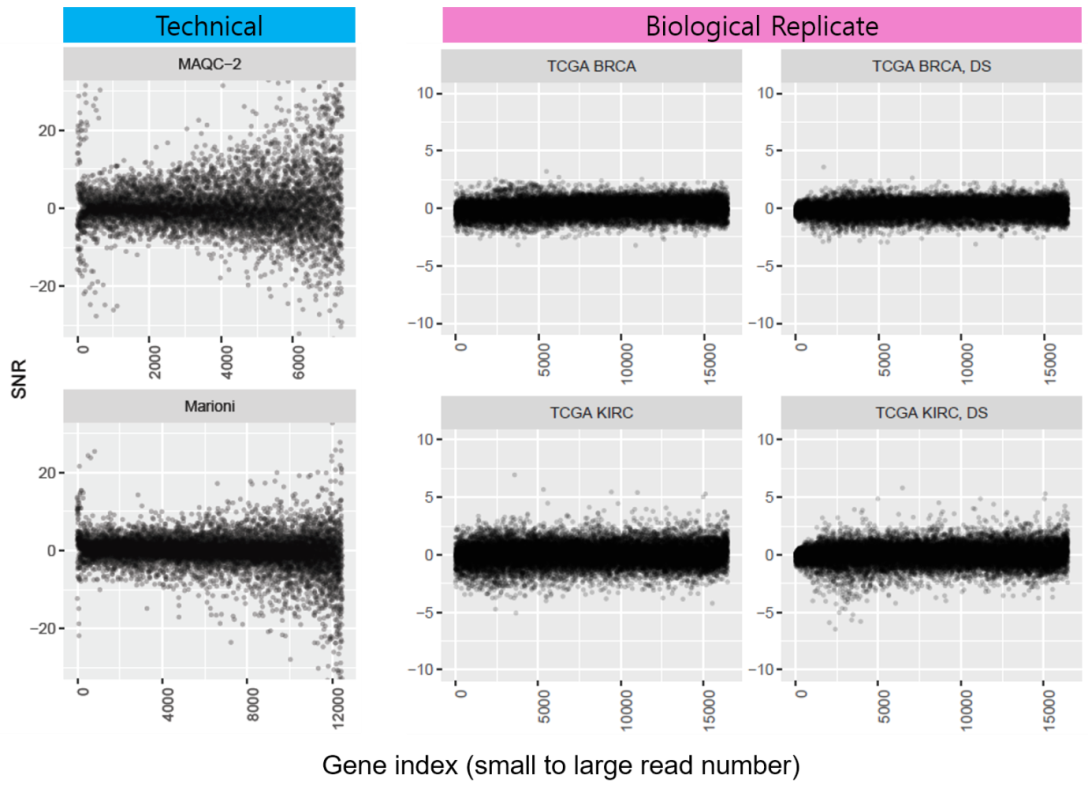


**Figure S1** The signal-to-noise patterns for two technical and biological replicate data for the voom (TMM) transformed data. The overall patterns were similar to the results for DESeq median normalized count data except the large variations for the small counts in the technical replicate data.

**
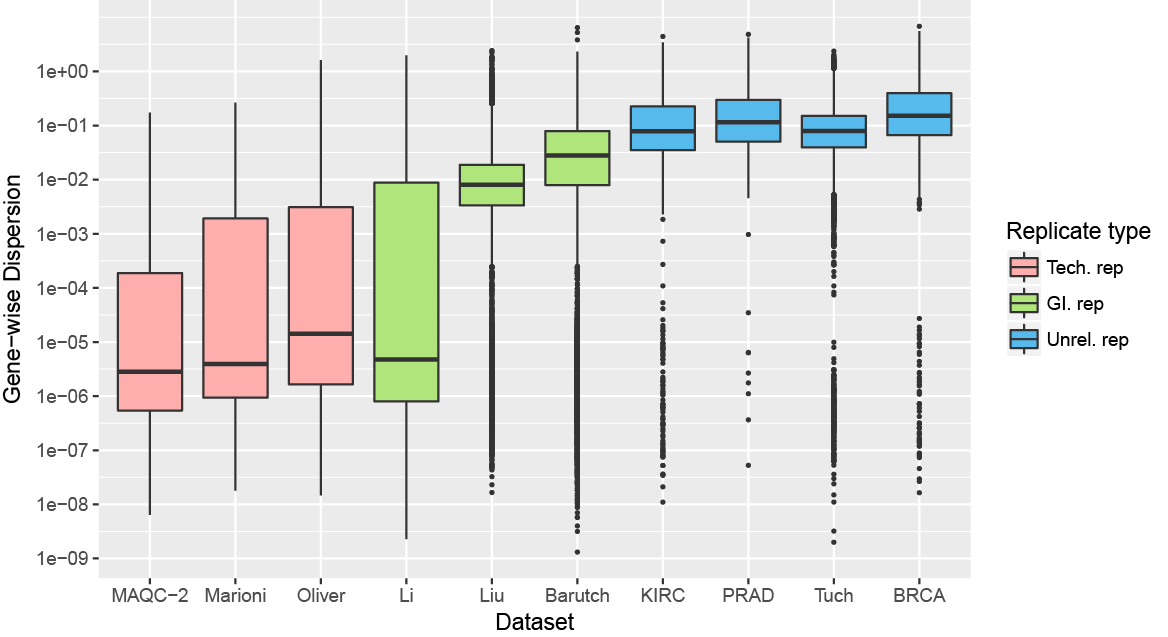
**

**Figure S2** The distribution of dispersions estimated using the naïve LRT method for the ten RNA-seq datasets analyzed in the main text.

(a) (b)

**
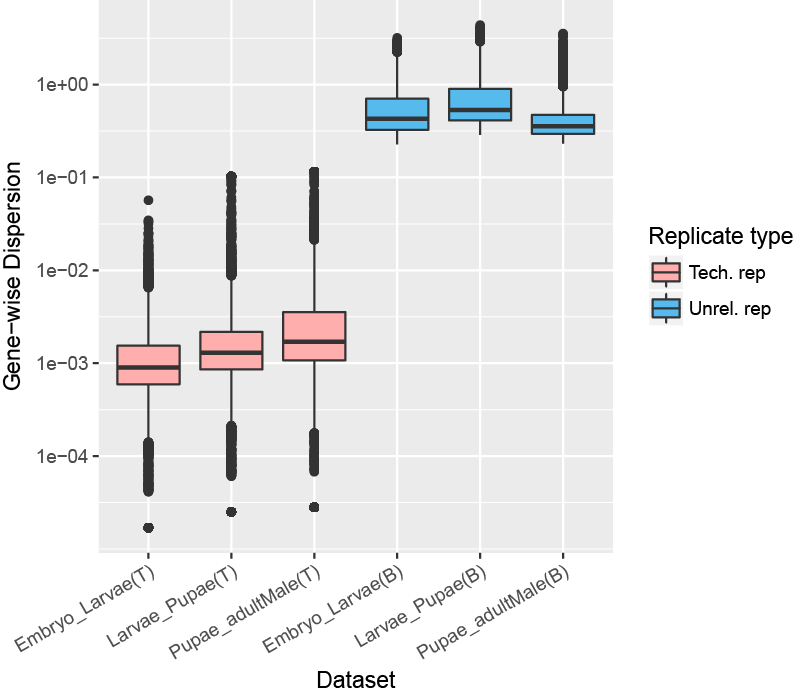

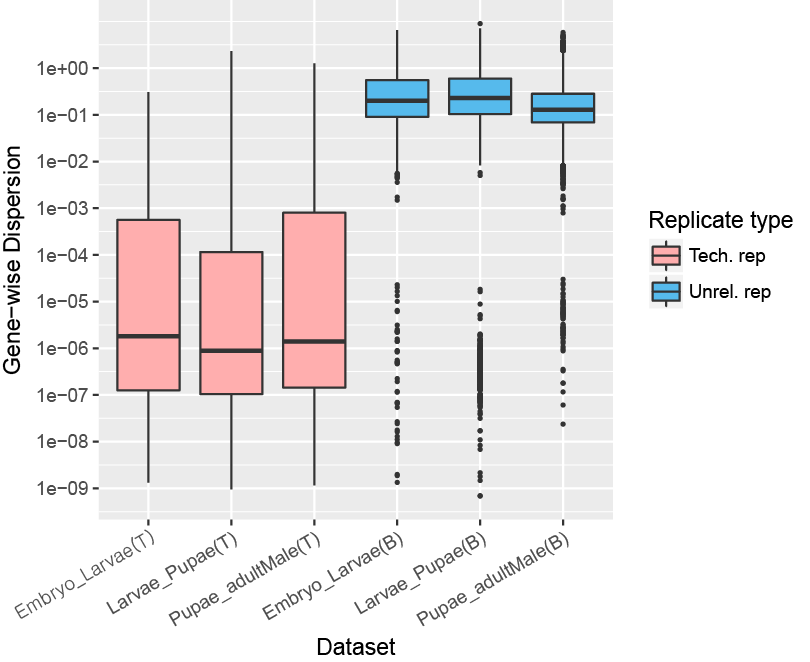
**

**Figure S3** The distribution of dispersions estimated using (a) edgeR and (b) naïve LRT for the six ModencodeFly RNA-seq data (Table 1).

**
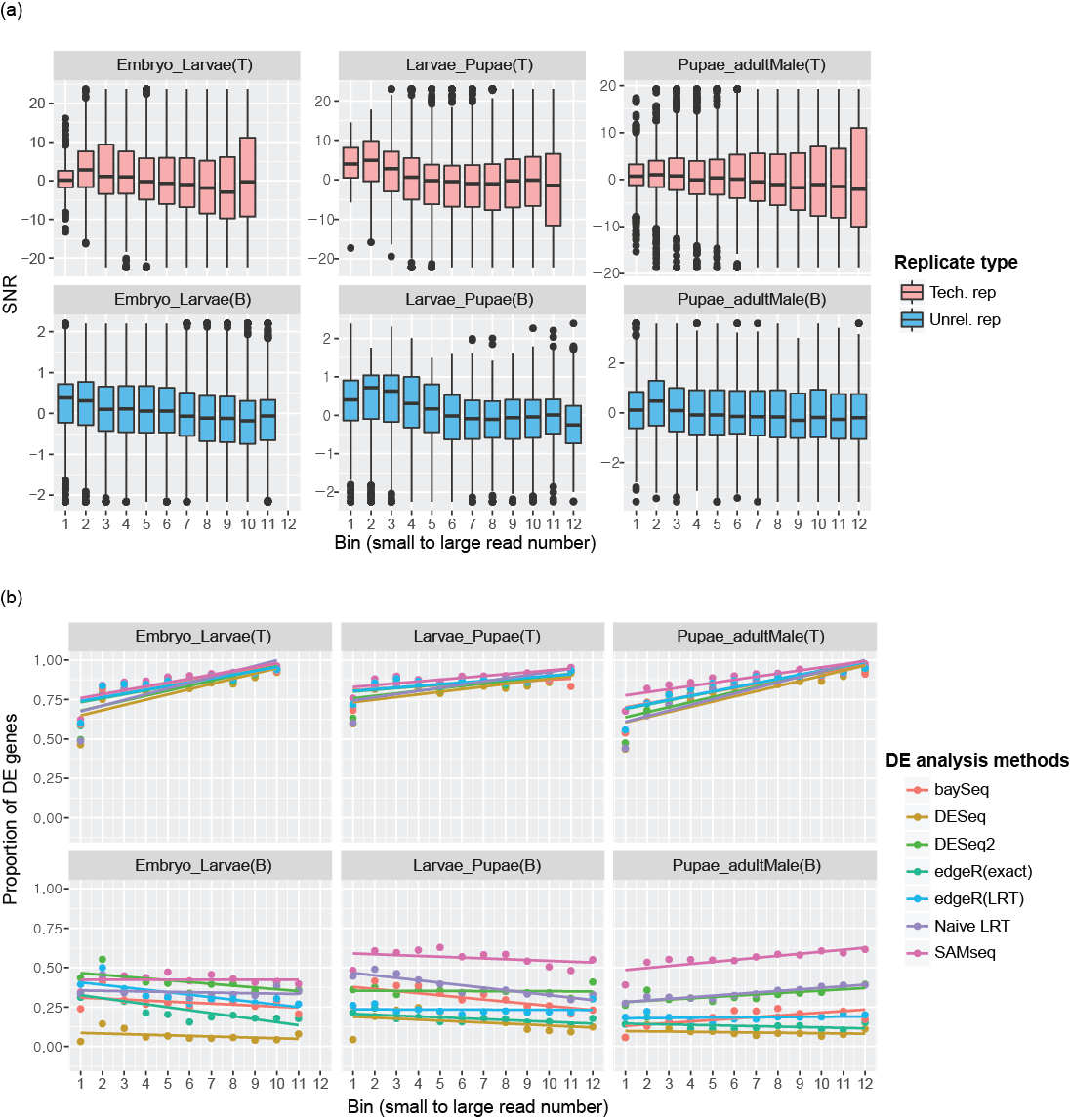
**

**Figure S4** Read count bias for the ModencodeFly datasets. For three pairs of the consecutive developmental conditions between late_embryo, larvae, Pupae and adult_Male, (a) the boxplots of SNRs are depicted against the read counts for technical replicate (first line) and unrelated replicate (second line) data. Each bin contains 1000 genes. (b) The proportions of DE genes in each bin were plotted for each dataset. The DE genes were obtained by using publicly available R packages.
